# Early prediction of putamen imaging features in HIV-associated neurocognitive impairment syndrome

**DOI:** 10.1186/s12883-021-02114-x

**Published:** 2021-03-09

**Authors:** Yu Qi, Man Xu, Wei Wang, Yuan-Yuan Wang, Jiao-Jiao Liu, Hai-Xia Ren, Ming-Ming Liu, Rui-Li Li, Hong-Jun Li

**Affiliations:** 1 Department of Radiology, Beijing Youan Hospital, Capital Medical University, No.8 Xi Tou Tiao Youanmen Wai, Fengtai District, Beijing, 100069 China; 2 Information and Communication Engineering Department Beijing University of Posts and Telecommunications, Beijing, China; 3 Department of Radiology, Beijing Second Hospital, Beijing, China; 4 Physical Examination Center, Cang zhou Central Hospital, Cang zhou, China

**Keywords:** HIV, Asymptomatic neurocognitive impairment, Putamen, Radiomics

## Abstract

**Background:**

To explore the correlation between the volume of putamen and brain cognitive impairment in patients with HIV and to predict the feasibility of early-stage HIV brain cognitive impairment through radiomics.

**Method:**

Retrospective selection of 90 patients with HIV infection, including 36 asymptomatic neurocognitive impairment (ANI) patients and 54 pre-clinical ANI patients in Beijing YouAn Hospital. All patients received comprehensive neuropsychological assessment and MRI scanning. 3D Slicer software was used to acquire volume of interest (VOI) and radiomics features. Clinical variables and volume of putamen were compared between patients with ANI and pre-clinical ANI. The Kruskal Wallis test was used to analysis multiple comparisons between groups. The relationship between cognitive scores and VOI was compared using linear regression. For radiomics, principal component analysis (PCA) was used to reduce model overfitting and calculations and then a support vector machine (SVM) was used to build a binary classification model. For model performance evaluation, we used an accuracy, sensitivity, specificity and receiver operating characteristic curve (ROC).

**Result:**

There were no significant differences in clinical variables between ANI group and pre-clinical-ANI group (*P*>0.05). The volume of bilateral putamen was significantly different between AHI group and pre-clinical group (*P*<0.05), but there was only a trend in the left putamen between ANI-treatment group and pre-clinical treatment group(*P* = 0.063). Reduced cognitive scores in Verbal Fluency, Attention/Working Memory, Executive Functioning, memory and Speed of Information Processing were negatively correlated with the increased VOI (*P*<0.05), but the correlation was relatively low. In diagnosing the ANI from pre-clinical ANI, the mean area under the ROC curves (AUC) were 0.85 ± 0.22, the mean sensitivity and specificity were 63.12 ± 5.51 and 94.25% ± 3.08%.

**Conclusion:**

The volumes of putamen in patients with ANI may be larger than patients with pre-clinical ANI, the change of the volume of the putamen may have a certain process; there is a relationship between putamen and cognitive impairment, but the exact mechanism is unclear. Radiomics may be a useful tool for predicting early stage HAND in patients with HIV.

## Background

When the human immunodeficiency virus (HIV) enters the human body, it can invade the central nervous system (CNS) in addition to attacking the immune system. The virus can infect the CNS in the early stage of infection and the viral RNA can be detected in cerebrospinal fluid within 8 days of HIV infection [1]. After breaking and penetrating the blood brain barrier (BBB), HIV can infect microglial cells and astrocytes [2–8]. The microglial cells undergo macrophage-like changes before secreting a variety of inflammatory factors which can activate more microglia, causing broader damage and direct or indirect injury to the neurons [6, 9, 10]. Astrocytes may serve as potential viral reservoirs in CNS; they can also secrete a neurotoxic regulatory protein called TAF which can damage the CNS [11].

Sustained damage caused by HIV can lead to multiple cognitive impairments in patients known as HIV-related neurocognitive disorders (HAND). HAND is classified into three levels according to severity: asymptomatic neurocognitive impairment (ANI), mild neurocognitive disorder (MND) and HIV-associated dementia (HAD) [12, 13].

15–50% of HIV+ patients live with HAND [12, 14]. HAD, a subcortical dementia and the most severe type of HAND, is relatively rare due to the widespread use of antiretroviral therapy (ART) [15]. ANI is the most common type of HAND: a multi-center study in the USA showed that ANI patients account for 70% of HAND patients [14];a smaller multi-center study revealed that HAND patients accounted for 37.1% of HIV+ patients in China, among which 60% were ANI patients and only 12% were HAD patients [16]. Although the degree of the neurocognitive decline is slow, ANI eventually deteriorates into MND and even into HAD [17]. The causes of the neurocognitive dysfunction are multifactorial, including factors such as age, education, cardiovascular diseases and whether ART is being taken. Therefore, clinical attention should be paid to screening ANI patients and taking active treatment and intervention measures [18].

The neuropsychological test is the golden standard of diagnosing ANI, but it is time-consuming and uncooperative for some patients. Conventional imaging examination can only obtain morphological information which cannot detect the changes of brain structure in the early stage of HIV+ patients. Diffusion tensor imaging (DTI) and functional magnetic resonance imaging (fMRI) can, to a certain extent, reflect the changes of brain structure in HIV+ patients but post-processing of such imaging is cumbersome and it has limited clinical application. Radiomics, which is widely used in central nervous system diseases, can extract a large number of image features from radiological images, and transform the image data into exploitable feature space data which can better reflect the characteristics of diseases [19, 20].

While HIV can affect any part of the brain, such as the frontal cortex, the temporal cortex, the thalamus, the cerebellum, the basal ganglia region is particularly vulnerable, this area is a hot spot of viral replication [21–25]. An animal study of Chinese rhesus macaques showed that the basal ganglia had the highest detection rate of SIV [26]. The volume of the putamen is susceptible in the early stages of HIV infection, and shrinks further as the infection progresses [27–29].

In this study, we investigate differences in the volume of the putamen between ANI and pre-clinical ANI patients, and the relationship between VOI and the cognitive status scores. We then extracted the radiomics signatures of the putamen and evaluated whether radiomics signatures can predict ANI.

## Methods

### Patients

Between June 2015 to June 2019, 90 HIV patients (36 ANI and 54 pre-clinical ANI) who underwent MRI and comprehensive neuropsychological assessment in Beijing YouAn Hospital were enrolled in this study. This study was approved by the ethics committee of Beijing YouAn Hospital and informed consent was obtained from all patients or their relatives (guardians). The inclusion criteria were as follows: (1) Patients had to have been diagnosed as HIV infectors by institutions qualified to confirm HIV infection; (2) Patients must be in the preclinical stage of HIV infection (no symptoms or mild cognitive impairment, no definite positive signs); (3) Age had to be from 18 to 80 years. The exclusion criteria were as follows: (1) HIV/AIDS patients transmitted by drug abuse or mother-to-child vertical transmission; (2) Anxiety, depression, alcoholism, stimulant use, drug side effects, metabolic encephalopathy, vitamin B12 deficiency and drug interaction; (3) Central nervous system diseases: tumors, cerebrovascular diseases and other visible diseases on MRI(T1WI and T2-fluid attenuated inversion recovery (FLAIR)); (4) Patients with MR contraindications: pacemakers, defibrillators, implanted electronic systems, vascular clips, mechanical heart valves or cochlear implants.(5) Patients with a sudden onset of illness.

To assess the cognitive status of patients with HIV, all patients underwent a comprehensive neuropsychological assessment which included 6 cognitive domains and a report of cognitive difficulties in daily life. The neurocognitive evaluation surveys contained: fine motor skills (Grooved Pegboard Test), information processing speed (Trail Marking Test A, TMT- A), memory (Hopkins Verbal Learning Test, HVLT-R; Brief Visuospatial Memory Test, BVMT-R), abstraction and executive function (Wisconsin Card Sorting Tests, WCST-64), attention and working memory (Continuous Performance Test-Identical Pair, CPT-IP; Wechsler Memory Scale, WMS-III; Paced Auditory Serial Addition Test, PASAT) and verbal/language (Animal Verbal Fluency Test, AFT). Self-questionnaires of daily functioning were assessed using a short Activity of Daily Living scale.Raw scores for each test is converted to T-scores, which is adjusted for age, sex, and education level. T-scores on more tests in a cognitive domain were averaged.

ANI was assessed using the Frascati criteria [13]: patients with ANI were considered to have two or more cognitive domains impaired and the daily life unaffected.

Clinical and demographic data including age, sex, CD4 count, the ratio of CD4/CD8, duration of infection and duration of treatment were collected. The duration of HIV infection was described by the patient’s own account. The recent CD4+ counts and the ratio of CD4/CD8 were collected within 2 weeks of the MRI.

### MRI protocols

All patients with HIV received MRI scanning using a Siemens Trio 3.0 Tesla scanner. The structural images were acquired using axial T1WI, and T2-FLAIR was collected to check whether there were visible intracranial lesions. Scanning parameters are as follows: time repetition (TR) = 250 ms, time echo (TE) = 2.46 ms, flip angle = 9°, field of vision (FOV) = 256 × 224 mm2, acquisition matrix = 256 × 256,section thickness = 1 mm,section number = 176; T2-FLAIR:TR = 8000 ms, TE = 2370.9 ms, inversion time = 97 ms.

### Volume of interest (VOI) acquisition and feature extraction

**Fig. 1:**
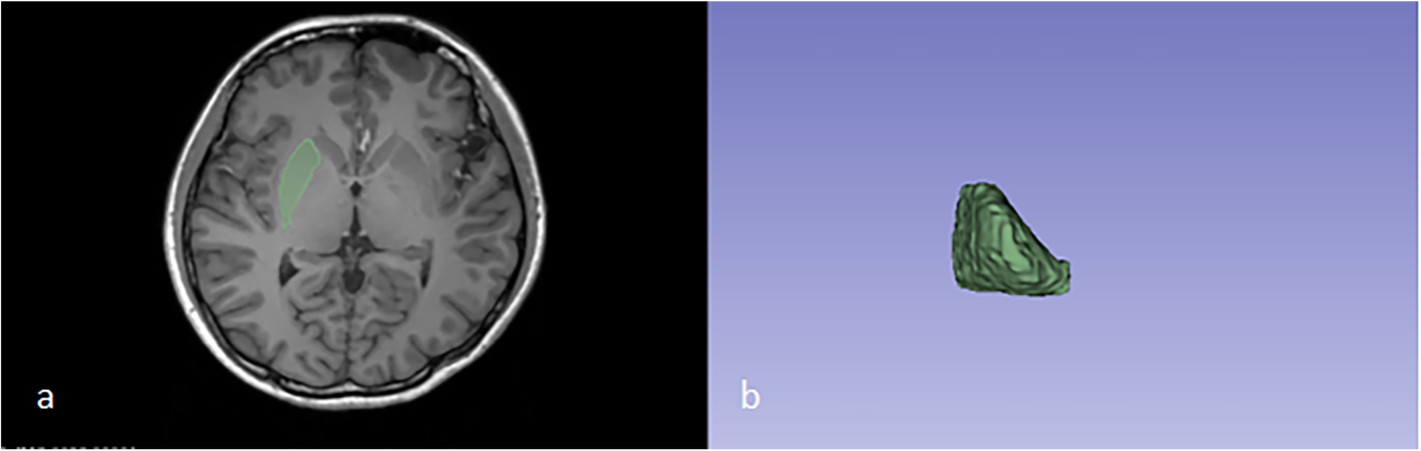
The process of segmentation. **a** VOIs were drawn in all axial MRI images. **b** VOI masks extracted from MRI scans

3D Slicer software (V4.11.0, http://download.slicer.org.) was used to acquire VOI and radiomics features(Fig. 1). We first obtained the bilateral putamen region by manual segmentation and then measured the volume; Secondly, SlicerRadiomics, an extension of 3D Slicer software was used to calculate the radiomics features of the bilateral putamen region, 851 features were extracted. Radiomics features are subdivided into the following classes: Shape-Based, First-Order Statistics, Gray Level Co-Occurrence Matrix, Gray Level Dependence Matrix, Gray Level Run Length Matrix, Gray Level Size Zone Matrix, Neighboring Gray Tone Difference Matrix.

### Statistical analysis

Analysis was performed using SPSS 22.0. All normally distributed variables were reported as mean ± SD, while non-normally distributed variables were reported as median (25th–75th percentile). A T-test was used for normally distributed data and the Mann-Whitney U Test was used for non-normally distributed data. A Chi-square Test was used for categorical data, and the Kruskal-Wallis Test was used for analysis of multiple comparisons between groups. Linear correlation was used to analyze the correlation between VOI and cognitive scores. *P* < 0.05 was considered statistically significant.

In order to reduce the impact of singular sample data, we used Z-score normalization to normalize all feature values, the calculation formula shown as eq.1-1. We also used principal component analysis (PCA) to reduce model overfitting and calculations, by selecting 25 principal components from the 851 features. After the feature reduction, we use a support vector machine (SVM) to construct a binary classification model taking data from PCA reduction as input data.

To evaluate the classification performance, we use a 10-fold cross-validation strategy to train and test the machine learning models. We also used accuracy, sensitivity, specificity and receiver operating characteristic (ROC) curve to evaluate model performance. The ROC curve was plotted in Python taking the true positive rate as the vertical axis and the false positive rate as the horizontal axis. We then calculated the area under the ROC curve (AUC): the higher the AUC value, the better the classification model performance.

1-1 $$ \chi {}_{\mathit{\operatorname{norm}}}=\frac{\chi -\mu }{\sigma } $$

Where, μ is the mean of all sample data and σ is the standard deviation of all sample data.

## Results

### Demographic and clinical characteristics

A total of 90 HIV patients were enrolled in this study with 36 patients in the ANI group, including 36 males and 0 female; there were 54 patients in the non-ANI group, including 53 males and 1 female. In the ANI group, 25 patients who were receiving stable ART treatment, the duration of treatment being was 16 months (6.5, 24); 11 patients received no treatment. In the pre-clinical ANI group, there were 37 patients who were receiving stable ART treatment, the duration of treatment time was 2 months (12.5,47); 17 patients were receiving no treatment.

There were 58 patients with complete clinical data, including 24 in the ANI group and 34 in the pre-clinical ANI group. The clinical and demographic characteristics are listed in Table 1. There were no significant differences in age, sex, CD4 count, CD4/CD8 ratio, duration of infection or duration of treatment between the ANI and pre-clinical ANI groups (*P*>0.05).

**Table 1:** Clinical and demographic data of study participants

Items	ANI(24)	pre-clinical ANI(34)	z/ x^2^	*p*-value
Age	30.54 ± 5.183	32.79 ± 7.176	1.388	0.171
Sex	24	34	–	1.000
CD4(cells/ml)	433.8 ± 186.19	500.5 ± 128.23	1.519	0.137
CD4/CD8	0.49(0.29, 0.73)	0.54(0.45, 0.65)	−0.869	0.385
Duration of infection (month)	19(9, 40)	24(9, 48)	−1.114	0.265
` duration of treatment time	16.0(6.3, 24.8)	20.0(9.8, 47.0)	−1.367	0.172

### VOI analysis between groups

**Fig. 2:**
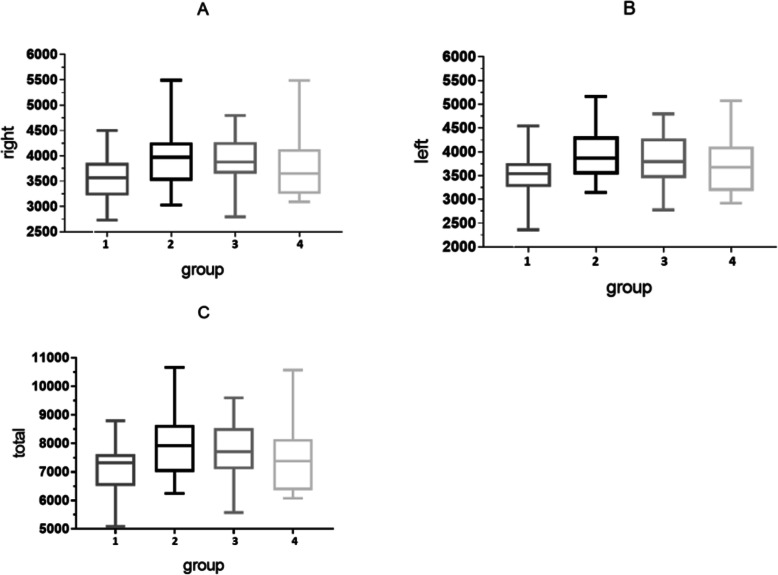
The relationship of the putamen between groups. *Fig. 2:1 = ANI-treatment group, 2 = ANI-treatment naive group,3 = non-ANI treatment group,4 = non-ANI treatment naive group. **a**:multiple comparisons between groups in the right putamen; **b**:multiple comparisons between groups in the left putamen; **c**:multiple comparisons between groups in the bilateral putamen

The volume of the bilateral putamen differed significantly between the ANI group and pre-clinical ANI group. The volume of putamen of the ANI group was larger than that of pre-clinical ANI group (Table 2). However, when consideration was given to the treatment, there was only a trend in the left putamen between the ANI-treatment group and pre-clinical ANI treatment group (*P* = 0.063, Fig. 2, Table 3).

**Table 2:** The differences in the volume of putamen between ANI and pre-clinical ANI

VOI (mm3)	ANI(36)	pre-clinical ANI(64)	*p*-value
Right	3972.06(3536.22,4234.79)	3639.22(3229,62,3944.64)	0.019
Left	3933.14 ± 542.975	3605.19 ± 501.632	0.005
Total	7761.96(7030.46, 8550.17)	7328.04(6469.96, 7856.84)	0.014

**Table 3:** The *P*-value of the volume of putamen between different groups

putamen	*p*-value between groups					
	1–2	1–3	1–4	2–3	2–4	3–4
Left	1.000	0.063	0.647	0.934	1.000	1.000
Right	1.000	0.157	1.000	1.000	1.000	1.000
Total	1.000	0.101	1.000	0.680	1.000	1.000

1 = ANI-treatment group, 2 = ANI-treatment naive group, 3 = non-ANI treatment group, 4 = non-ANI treatment naive group. *P* value between groups. Kruskal Wallis test was used. *P*<0.05

### Correlations between VOI and cognitive performance and age for HIV+ patients

Table 4 shows the correlation between VOI and cognitive performance. Reduced cognitive scores in Verbal Fluency, Attention/Working Memory, Executive Functioning, Memory and Speed of Information Processing were negatively correlated with the increased VOI; correlation was, however, relatively low. There was no correlation between Fine Motor Skills and VOI.

**Table 4:** The correlation between VOI and cognitive status scores

Cognitive	right		left		total	
Status/age	r	P	r	p	r	p
VF	−0.244	0.020	−0.336	0.002	−0.289	0.006
A/WM	−0.243	0.021	−0.262	0.013	−0.260	0.013
EF	−0.245	0.020	−0.233	0.027	−0.247	0.019
M (LDR)	−0.267	0.011	−0.256	0.015	−0.270	0.010
SIP	−0.227	0.032	−0.218	0.039	−0.230	0.030
FMS	−0.115	0.282	−0.087	0.414	−0.104	0.328

*The correlation coefficients and significance results were calculated by the pearson correlation. VF Verbal Fluency, A/WM Attention/Working Memory, EF Executive Functioning, SIP Speed of Information Processing,FMS,Fine Motor Skills

### Predictive performance of the radiomics features

**Fig. 3:**
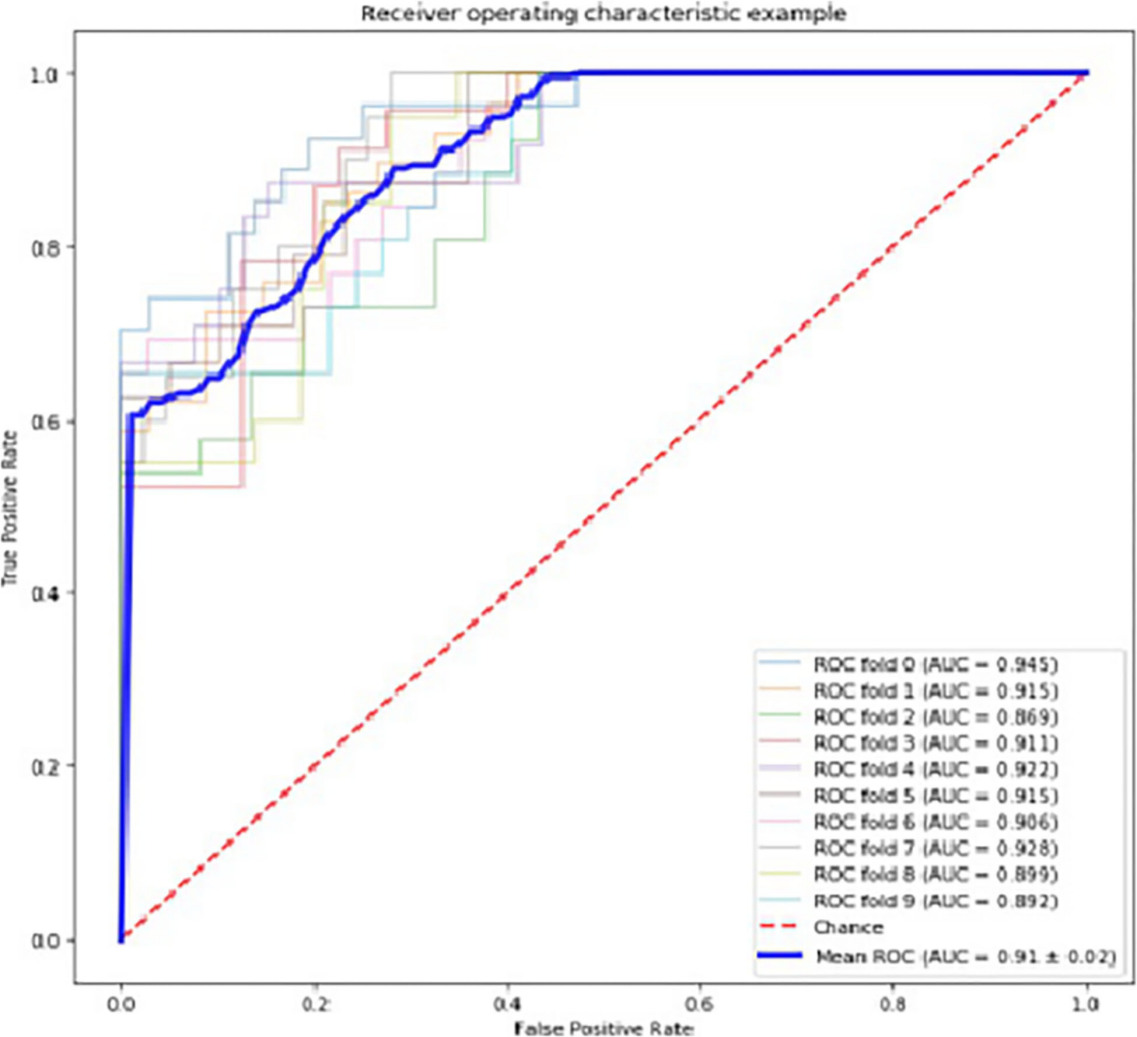
ROC curves analysis of radiomics signatures for differentiating ANI

Twenty-five principal components were ultimately selected through PCA. In diagnosing the ANI from pre-clinical ANI, the mean AUCs were 0.85 ± 0.22(Fig. 3), the mean sensitivity and specificity were 63.12 ± 5.51 and 94.25% ± 3.08%.

## Discussion

The basal ganglia is the earliest region to have inflammation of acute HIV infection [30]; many studies using different cohorts have shown changes in the volume and metabolism of the basal ganglia including putamen [30]. Injury of the putamen also occurs in other disease such as multiple sclerosis, Alzheimer’s disease and Type 1 Diabetes [31–33]. The neostriatum, including the putamen and caudate nucleus, receives afferent fibers from the cerebral cortex (mainly the frontal and parietal lobes) as well as recurrent fibrous connections to the old striatum, substantia nigra, and subthalamus. Therefore, an injured putamen may cause some dysfunctions. HIV-1 enters the CNS and starts replicating the virus which causes an inflammatory response leading to neurological disorders called HAND. Although use of ART has dramatically reduced the ratio of HAD [34], ANI and MND remain highly prevalent, particularly ANI. Immune activation and inflammation are still observed in patients with ART. Therefore, we chose the patient’s characteristics of putamen to analysis differences between ANI and pre-clinical ANI without considering the effect of treatment.

The neostriatum comprises the putamen and caudate nucleus. Our study shows that the volume of the bilateral putamen is related to the score of Verbal Fluency, Attention/Working Memory, Executive Functioning and Speed of Information Processing, but correlation is low. The putamen was thought to play a role in motor planning and control, but in recent years many studies shows that putamen also play a role in lexical, morphological syntactic and speech production processes [35–37]. Many studies have demonstrated that the functions of the anterior and posterior of the putamen are different [38–40]. The posterior portion links with the motor cortex while the anterior portion links with other cortexes. According to a meta-analysis [41], the left putamen has coactivations with many regions such as the bilateral middle frontal gyrus (MFG), bilateral precentral gyrus (PG), bilateral superior temporal gyrus (STG). The left MFG is associated with verbal fluency [42]. Other studies have shown that the superior parietal lobule (SPL) and superior frontal gyrus (SFG), which have a connection with the putamen, are associated with manipulation of information in the working memory [43, 44]. The right putamen might also have a secondary role [41]. Therefore, changes in the volume of the putamen may indicate damage to it which could lead to a decline in some cognitive functions. The dopamine system in the striatum plays a role in cognitive control and striatum dysfunction can impair some cognitive processes. In patients with X-linked dystonia parkinsonism (XDP), for example, cells are lost in the striatum in the early stage and the striatum is degenerative, the stability of monitor behaviors decreases, and the sensory memory process is reduced [45–47]. Whether this contributes to neurocognitive disorders in patients with HIV is unclear. Therefore, more studies into the putamen and cognitive disorders should be undertaken to better understand this mechanism.

A study showed that the volume of putamen reduced in patients with chronic HIV infection despite receiving stable on ART (over 1 year) without changes [48]. Another study showed that compared to HIV-uninfected individuals, the volume of putamen had a trend-level moderate reduction in patients with primary HIV infection (PHI) [28]. However, to date there has been no study to investigate the volume of putamen in groups based on the cognitive status of HIV patients. In the current study, we examined the volume of putamen in patients with ANI and non-ANI, and showed that volume of bilateral putamen in patients with ANI is larger than that in patients with non-ANI. In the CNS of HIV-infected patients, macrophages and microglia may play an important role. Monocytes in the blood pass through the damaged BBB and differentiate into perivascular macrophages; they may then release a virus that can infect resident brain macrophages and microglia [49]. Once activated, the volume of microglia, which are usually distributed around the gray matter neurons and near small blood vessels, increases and amoeboid changes occur [50]. The contribution of microglia to neurocognitive deficit may be the increase in proinflammatory chemokines and cytokines [10]. The basal ganglia is the earliest area where inflammation occurs [30], and HIV replication preferentially occurs in perivascular macrophages in the basal ganglia [51]. These pathological changes may lead to changes in the volume of the putamen. However, our results are contrary to previous results, which may indicate that there is a certain process of change in putamen volume in patients with HIV. Furthermore, our study shows a trend of growth in the left putamen in ANI-treatment group in comparison to the non-ANI treatment group. This result suggests that the change in volume may be related to the effect of ART drugs. However, controversy exists around the effect of drugs on CNS. Several studies have shown that ART can improve patients’ cognitive performance and slow the rate of brain tissue atrophy [52, 53]; however, patients with a controlled viral load had improved cognitive status after discontinuing treatment [14, 54]. While it is true that ART is unable to eliminate the latent reservoir and the continued replication in CNS [7, 55], and it can also damage mitochondrial function and other structures, whether these lead to volume changes is unclear.

Traditional radiological examination can only obtain morphological, signal characteristics or density information of diseases. Some diseases cannot be diagnosed and differentiated through traditional radiological examination. In addition, the accuracy of the diagnosis depends on the radiologists’ clinical experience, learning experience, etc. Radiomics transforms traditional image data into characteristic spatial data that can be mined, and carries out high-throughput quantitative analysis to comprehensively evaluate information including tissue morphology, cell molecules, gene inheritance, etc. [56, 57] Radiomics has become increasingly mature and has been applied in multiple systems of disease diagnosis in the field of medicine. Radiomics has been used in cancers such as glioma, hepatocellular carcinoma, breast cancer and other tumors [58–60]. Recently, radiomics has also been applied to evaluation of the cognitive status in some diseases; for instance, Betrouni N [19] found that texture features of images can predict cognitive impairment after stroke; Zhou [61] used radiomic biomarkers to predict patients with mild cognitive impairment (MCI) progressing to Alzheimer’s disease (AD), Yupeng Li [20] considered radiomics features can be applied into predicting AD from MCI, the accuracy was 80.0, 93.3 and 86.6% to distinguish MCI-to-AD fast and slow converter using linear, polynomial and sigmoid kernels. However little study has been undertaken in patients with HIV. Compared with HIV-uninfected individuals, the brain volume and cognitive function of HIV-infected patients declines despite the use of ART; but ART can delay or even reverse the thickness of brain cortex in HIV-positive patients compared with antiretroviral-naïve patients, and there was no further decline in neuropsychological cognitive impairment in HIV-positive patients with ART [25, 62]. Therefore, screening ANI patients and taking active treatment and intervention measures is important. In our study 851 features were extracted and PCA was then used to further select principal components which led to the final selection of 25 principal components. After the dimension reduction, the data obtained from dimension reduction were modeled by SVM. In our study, PCA was chosen because it only needs to measure the amount of information by variance and is not affected by factors other than the data set. Futher more, it can eliminate the mutual influence factors between the original data components and reduce data noise. The feature dimension in our study is greater than the number of samples, SVM is suitable for this kind of scenario, SVM uses a part of support vectors to make hyperplane decisions and does not rely on all data, so it had higher classification accuracy and stronger generalization ability for small data sets. Finally, the results show that accuracy and sensitivity were 63.12 ± 5.51 and 94.25% ± 3.08% respectively while the AUCs were 0.85 ± 0.22. The results show that this model has a certain screening effect on ANI but the accuracy was relatively poor. There may be two possible reasons for this result: firstly, the insufficient sample size in our study; and secondly, the sample size between ANI and pre-clinical ANI was imbalanced. Our results show that radiomics may be one useful approach that can screen ANI from patients with HIV.

There were some limitations in our study. Firstly, a few patients were enrolled, and the proportion of ANI patients was relatively low, which may affect the prediction effect of the model; further analysis with larger samples and a longitudinal study should be done. Secondly, 3D slicer software was used to extract the radiomics features and to acquire VOI by manual segmentation; there will be some errors in the later steps. Thirdly, our study only discussed patients with ANI and those with pre-clinical ANI, MND and HAD were not discussed. Future research needs to address this issue.

## Conclusion

In summary, the volumes of the putamen in patients with ANI may be larger than patients with pre-clinical ANI so the change of the volume of the putamen may have a certain process; there is a relationship between the putamen and cognitive impairment, but the exact mechanism of this is unclear; radiomics may be a useful tool for predicting HAND in patients with HIV. In the future, we will focus on a large sample and longitudinal study on analyzing the structure and function networks of HIV patients by targeting the putamen and multi-moding data to study radiomics in predicting HAND by adopting different models. Further research on this technique will provide important evidence for early clinical intervention and improve the quality of life of patients with HIV.

## Data Availability

The datasets used in this manuscript will be available from the corresponding author on reasonable request.

## Abbreviations

ANI: Asymptomatic neurocognitive impairment
VOI: Volume of interest
PCA: Principal component analysis
SVM: Support vector machine
ROC: Receiver operating characteristic curve
AUC: Area under the ROC curve
HIV: Human immunodeficiency virus
CNS: Central nervous system
BBB: Blood brain barrier
HAND: HIV-related neurocognitive disorders
MND: Mild neurocognitive disorder
HAD: HIV-associated dementia
ART: Antiretroviral therapy
DTI: Diffusion tensor imaging
fMRI: Functional magnetic resonance imaging
FLAIR: Fluid attenuated inversion recovery
TR: Time repetition
TE: Time echo
FOV: Field of vision
MFG: Bilateral middle frontal gyrus
PG: Precentral gyrus
STG: Bilateral superior temporal gyrus
SPL: Superior parietal lobule
SFG: Superior frontal gyrus
XDP: X-linked dystonia parkinsonism
PHI: Primary HIV infection
MCI: Mild cognitive impairment
AD: Alzheimer’s disease
